# Phosphorus MRS of healthy human spleen

**DOI:** 10.1002/nbm.4779

**Published:** 2022-06-15

**Authors:** Jan Weis, Maysam Jafar, Per Liss

**Affiliations:** ^1^ Department of Medical Physics Uppsala University Hospital Uppsala Sweden; ^2^ Philips Nordic Solna Sweden; ^3^ Section of Radiology, Department of Surgical Sciences University Hospital Uppsala Sweden

**Keywords:** 3 T, feasibility, healthy spleen, ISIS, phosphorus MRS

## Abstract

Phosphorus (^31^P‐) MRS in vivo enables detection and quantification of important phosphorus‐containing metabolites in biological tissues. ^31^P‐MRS of the normal spleen is challenging due to the relatively small volume and the larger distance between the spleen and surface coil. However, reference spectra of the healthy spleen are invaluable in studies of splenic malignancies and benign causes of splenomegaly, as well as in the study of its physiology. The purpose of this work was to investigate the feasibility of localized ^31^P‐MRS of healthy spleen in situ in a clinically acceptable measurement time using a clinical 3 T MR scanner. In this work, ^31^P spectra of five healthy volunteers were measured using single‐voxel image‐selected in vivo spectroscopy (ISIS). The measurement sequence was augmented by broadband proton decoupling and nuclear Overhauser effect enhancement. It is demonstrated that localized ^31^P‐MRS of the spleen in situ using single‐voxel ISIS is feasible on a clinical 3 T scanner in a clinically acceptable acquisition time. However, results have to be corrected for the transmitter excitation profile, and chemical shift displacement errors need to be taken into consideration during placement of the volume of interest. Results presented here could be used as a reference in future studies of splenomegaly caused by haematological malignancies.

## Graphical abstract

Phosphorus MRS of healthy human spleen in situ using single‐voxel ISIS is feasible on a 3 T scanner in a clinically acceptable acquisition time. Results should be corrected for transmitter excitation profile, and chemical shift displacement errors need to be taken into consideration during placement of the voxel.

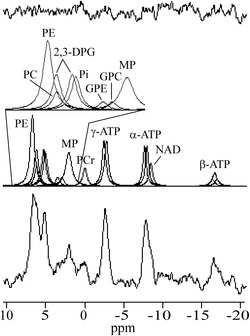

**Abbreviations**
^1^H: proton
^31^P: phosphorus
AMARES: advanced method for accurate, robust and efficient spectral fitting
AP: anterior–posterior
ATP: adenosine triphosphate
CRLB: Cramér–Rao lower bound
CSDE: chemical shift displacement error
DPG: diphosphoglycerate
DRESS: depth‐resolved surface‐coil spectroscopy
FH: feet–head
FID: free induction decay
GPC: glycerol‐3‐phosphocholine
GPE: glycerol‐3‐phosphoethanolamine
ISIS: image‐selected in vivo spectroscopy
MP: membrane phospholipid
MRSI: magnetic resonance spectroscopic imaging
NAD: nicotinamide adenine dinucleotide
NOE: nuclear Overhauser effect
PCr: phosphocreatine
PDE: phosphodiester
PE: phosphoethanolamine
P_i_
: inorganic phosphate
PME: phosphomonoester
RL: right–left
SNR: signal‐to‐noise ratio
VOI: volume of interest

## 1 INTRODUCTION

Phosphorus (^31^P‐) MRS enables detection and quantification of various phosphorus‐containing metabolites in biological tissues. Furthermore, it provides assessment of intracellular pH, cerebral Mg^2+^, and the kinetics of chemical reactions of energy metabolism.^1^, ^2^ Compared with proton (^1^H) spectroscopy, ^31^P‐MRS requires relatively large voxel sizes and longer measurement times because of lower sensitivity and lower concentrations of ^31^P metabolites in vivo. Therefore, most studies have been performed with large, homogeneous tissues (e.g., brain, liver, muscles) whereby a larger volume of interest (VOI) can be chosen and the distance between the ^31^P transmit–receive surface coil and the VOI is relatively small. The majority of experiments were performed using image‐selected in vivo spectroscopy (ISIS),^3^, ^4^, ^5^, ^6^ depth‐resolved surface‐coil spectroscopy (DRESS),^7^ or free induction decay (FID) 3D magnetic resonance spectroscopic imaging (MRSI) localization sequences.^8^, ^9^, ^10^ These are FID‐based methods with a very short acquisition delay, which results in signal enhancement from short *T*_2_ phosphorus metabolites and the minimization of *J* modulations on the acquired spectrum. DRESS and 1D ISIS sequences are slab selective and use the sensitive volume of the transmit–receive surface coil to define the VOI in the other two dimensions. Slab selective methods can be combined with MRSI localization.^11^, ^12^ Their main drawback is the less accurate VOI selection caused by the sensitive volume of the surface coil. Contamination of the acquired spectra by signals from neighbouring tissues is a consequence of such VOI selection. Another drawback is the need for transmit–receive coils of different sizes when a change of VOI size is necessary. The DRESS technique is robust to motion artefacts, which is an important feature during dynamic examinations.^13^ FID‐based 3D MRSI localization avoids slab selection by including the whole sensitive volume of the surface coil in the 3D MRSI field of view. The main disadvantage of MRSI localization is signal contamination (signal bleeding) from adjacent voxels due to the small fraction of *k*‐space covered in the measurement.^14^ An alternative to the 1D ISIS slab selection is 2D ISIS bar or 3D ISIS single‐voxel localization. ISIS methods are prone to motion artefacts because they combine two (1D ISIS), four (2D ISIS), or eight (3D ISIS) FIDs acquired in different excitation cycles. Furthermore, ISIS sequences use adiabatic excitation pulses because they are relatively insensitive to the *B*_1_ inhomogeneity of the transmit–receive surface coil. Unfortunately, adiabatic slice selective inversion and non‐slice selective detection *B*_1_ pulses have limited bandwidth. This results in large chemical shift displacement errors (CSDEs) and the dependence of the excitation profile on distance between the surface coil and the VOI.^5^

Compared with ^31^P‐MRS of the liver, brain, or muscles, spectroscopy of the healthy spleen is challenging due to its smaller volume and increased distance from the transmit–receive surface coil. Only a few reports of ^31^P spectra of the human spleen have been published so far.^15^, ^16^, ^17^ In 1989, Smith et al. reported ^31^P‐MRS of the human spleen at 1.5 T.^15^ Experiments were performed using 1D MRSI without ^1^H decoupling or nuclear Overhauser effect (NOE) enhancement. The sensitive volume of the surface coils was combined with 1D spectroscopic imaging where the third dimension was encoded by a phase gradient perpendicular to the surface coil. The authors demonstrated that spectra of malignant disorders had an increased level of phosphomonoester (PME) intensities compared with those of healthy volunteers. Negendank et al.^16^ and Arias‐Mendoza et al.^17^ performed ^31^P‐MRS of normal spleen and benign splenomegaly. ^1^H decoupled and NOE enhanced spectra were acquired on a 1.5 T scanner using an FID‐based 3D MRSI sequence.^8^, ^16^ The authors did not observe differences between normal spleen and benign splenomegaly. PME in splenic NHL (non‐Hodgkin lymphoma) was found to be more intense compared with normal spleen.

The main purpose of this work was to repeat ^31^P‐MRS experiments on healthy volunteers. The second purpose was to investigate the feasibility of ^31^P‐MRS of healthy spleen in situ in a clinically acceptable measurement time on a clinical 3 T scanner.

## 2 METHODS

### 2.1 Human subjects

Five healthy volunteers (two females and three males) were recruited for this study. The median age of the subjects was 37 years (range 34–46). The local institutional review board approved this study and written informed consent was obtained from each participant.

### 2.2 Data acquisition

^31^P spectra of the volunteers were acquired on a 3 T clinical MR scanner (Achieva dStream, Philips Healthcare, Best, the Netherlands) using a circular transmit–receive surface coil (diameter 140 mm) with manual tuning. Maximum *B*_1_ achievable at the centre of the coil was set to 60 μT. Balanced turbo field echo (BTFE) images were acquired in axial, sagittal, and coronal planes to guide planning of the voxel position. Sagittal and coronal images were oriented parallel and perpendicular to the surface coil, respectively. The voxel was selected using the 3D ISIS sequence augmented by broadband ^1^H decoupling and NOE enhancement. Measurement parameters were *T*_R_ 5000 ms, 512 scans, spectral bandwidth 3000 Hz, 2048 complex points, and net acquisition time 43 min. Magnetic field homogeneity was improved by pencil beam second order shimming. The whole‐body coil was used for imaging, decoupling and NOE enhancement. The 3D ISIS sequence was based on a simple eight acquisition scheme and all measurements were made with free breathing. A hyperbolic‐secant adiabatic pulse was used for excitation (pulse length 5.42 ms, excitation bandwidth 1.15 kHz). It should be noted that the excitation adiabatic hyperbolic‐secant pulse is not fully adiabatic. The excitation angle varies as a function of *B*_1_. Slice selections were performed using hyperbolic‐secant adiabatic full‐passage inversion pulses (pulse length 4.22 ms, bandwidth 2.95 kHz). NOE enhancement was accomplished with narrow band irradiation, mixing time 4100 ms, offset frequency from water line −100 Hz, and NOE *B*_1max_ 0.5 μT. WALTZ‐4 phase cycling broadband ^1^H decoupling pulses were applied with the offset frequency −100 Hz.

The transmitter excitation profile was measured using a spherical phantom (diameter 10 cm) containing 50 mM monopotassium phosphate (KH_2_PO_4_) water solution. The largest side of the voxel (30 × 50 × 50 mm^3^) was placed parallel with the surface coil. The ^31^P spectral intensity of the inorganic phosphate (P_i_) singlet was measured as a function of transmitter carrier frequency.

### 2.3 Spectrum processing

Spectrum processing was carried out using the AMARES (advanced method for accurate, robust and efficient spectral fitting) algorithm (the jMRUI—Java‐based magnetic resonance user interface—software package) without previous apodization of the FID.^18^ However, an exponential apodization corresponding to 8 Hz line broadening was applied for the purpose of illustration. The frequency axis was defined by placing the γ‐adenosine triphosphate (ATP) doublet at −2.53 ppm.^2^, ^19^ This spectral line was chosen because it was the most reliable to fit. It was assumed that the small dependence of γ‐ATP on intracellular pH does not play a substantial role for the group of healthy volunteers in the physiological range around 7.2. Initial positions of phosphoethanolamine (PE) at 6.77 ppm, the 2,3‐diphosphoglycerate doublet (DPG, 6.24 and 5.24 ppm), P_i_ (4.84 ppm), glycerol‐3‐phosphoethanolamine (GPE, 3.49 ppm), glycerol‐3‐phosphocholine (GPC, 2.94 ppm), and phosphocreatine (PCr, 0 ppm) were taken from previous publications.^2^, ^16^, ^19^, ^20^ 2,3‐DPG spectral lines of erythrocytes were included in the prior knowledge because the spleen contains around 30–40% blood volume.^16^ The best fit of membrane phospholipid (MP) signals was achieved by placing this broad line at 2.15 ppm. Positions of the spectral lines were estimated using AMARES. Soft constraints ±0.05 ppm were applied to the PE, 2,3‐DPG, GPE, GPC, MP, and PCr peak positions. The P_i_ line position was estimated without soft constraints. The position of the phosphocholine (PC) peak was fixed at −27.92 Hz with respect to PE.^2^, ^19^ Spectral line positions of γ‐, α‐, and β‐ATP were estimated using AMARES without soft constraints. The nicotinamide adenine dinucleotide (NAD) line was fixed at −41.61 Hz with respect to α‐ATP.^2^ The splitting of ATP multiplets was set to 16 Hz.^19^, ^21^ The amplitude ratios of doublets (2,3‐DPG, γ‐, α‐ATP) and triplets (β‐ATP) were fixed to 1:1 and 1:2:1, respectively. The linewidths of PE, PCr, and MP were constrained to the intervals 10–25 Hz and the linewidth of y‐ATP was constrained to 25–40 Hz, respectively. PC, 2,3‐DPG, P_i_, GPE, and GPC linewidths were set equal to that of PE.^2^, ^19^ The linewidth ratios of α‐ATP, NAD, and β‐ATP peaks were fixed with respect to γ‐ATP to 0.8, 1, and 1.4, respectively.^2^, ^19^ The relative phase of all spectral lines was set to zero. No baseline correction was needed. The zero‐ and first‐order phase corrections were estimated using AMARES. The first‐order phase correction was constrained to ±0.5 ms in the time domain and spectral lines were fitted using a Lorentzian lineshape. The spectra of all volunteers were phase and frequency corrected and added together. The PME (PE + PC), 2,3‐DPG, P_i_, phosphodiester (PDE: GPE + GPC), MP, and γ‐ATP spectral lines were used in quantifications, and α‐ATP, NAD, and β‐ATP intensities were excluded. The excluded peaks suffered from a severe reduction in intensity due to the transmitter excitation profile and to large CSDEs. Spectral intensities were expressed as a percentage fraction of summed PME, 2,3‐DPG, P_i_, PDE, MP, and γ‐ATP intensities. The PCr peak was not included in the summation because it originates from adjacent skeletal muscle. Since PC and P_i_ lines are overlapped by strong 2,3‐DPG peaks and PDE lines are at the noise level, only fractions of PME + 2‐DPG, 3‐DPG + P_i_, PDE + MP, and γ‐ATP were quantified. In addition, spectral intensity ratios to 3‐DPG + P_i_ and to γ‐ATP were evaluated for the sake of completeness. These values were not corrected for *T*_1_ and NOE effects because *T*_1_ quantification and NOE enhancement were not possible due to the long measurement times (2–3 h per volunteer). Furthermore, correction of γ‐ATP intensity for ATP content in the blood was not performed because of the less accurate blood content in a healthy human spleen (around 30–40%)^16^ and unreliable quantification of 2,3‐DPG intensities.

### 2.4 Statistics

The reported values are given as the mean ±1 SD.

## 3 RESULTS

**FIGURE 1:**
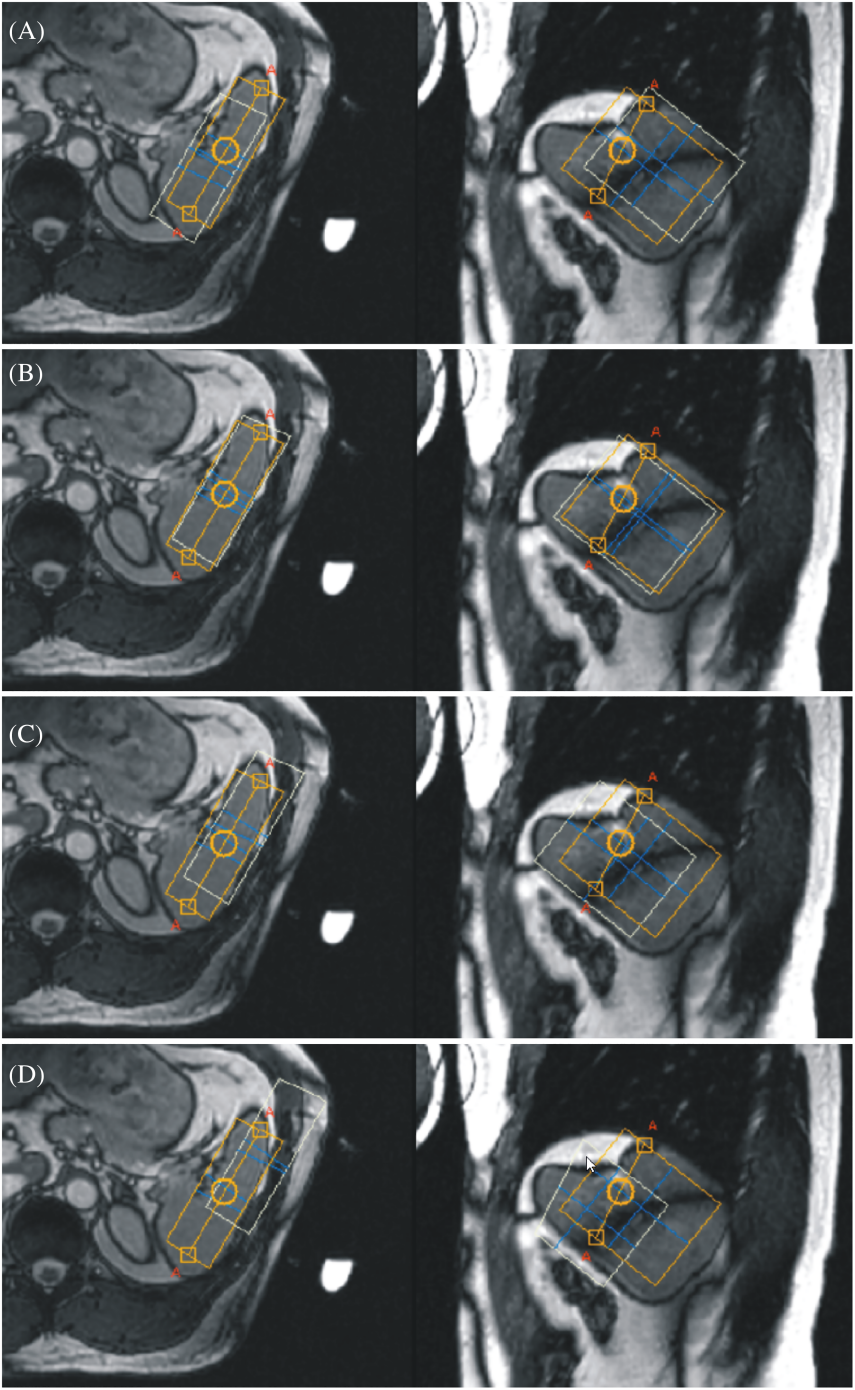
A typical voxel size (3 × 7 × 6 cm^3^) and position in axial and sagittal planes. A small water‐containing probe was attached to the centre of the coil on the outer side as a marker of coil position. Yellow rectangles show voxel positions corresponding to the carrier frequency of the transmitter. White rectangles depict voxel positions corresponding to PE (A), γ‐ATP (B), α‐ATP (C), and β‐ATP (D) spectral lines.

**FIGURE 2:**
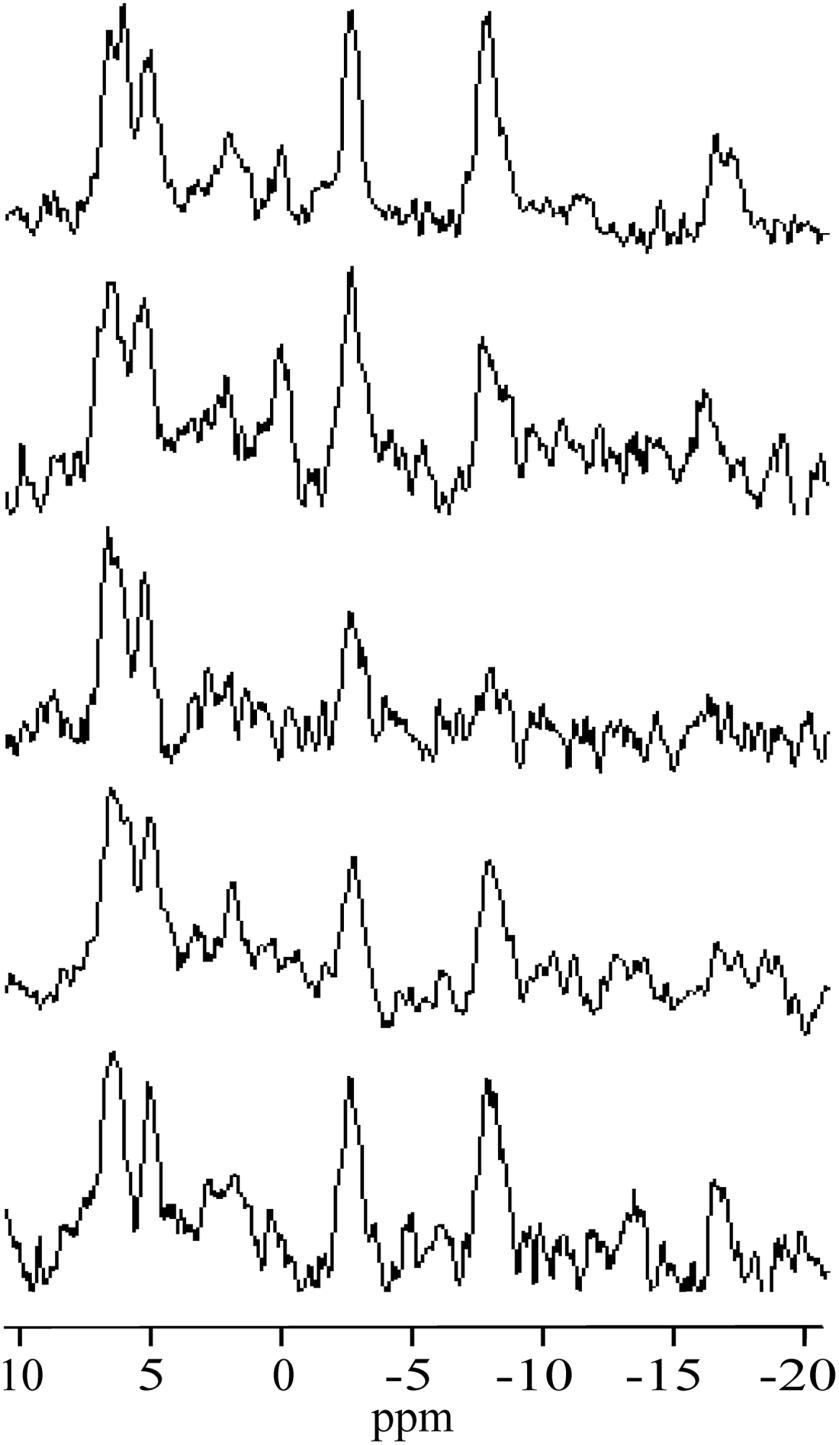
^31^P spectra of the spleens of five healthy volunteers. A Lorentzian apodization of the FIDs corresponding to 8 Hz line broadening was applied.

**FIGURE 3:**
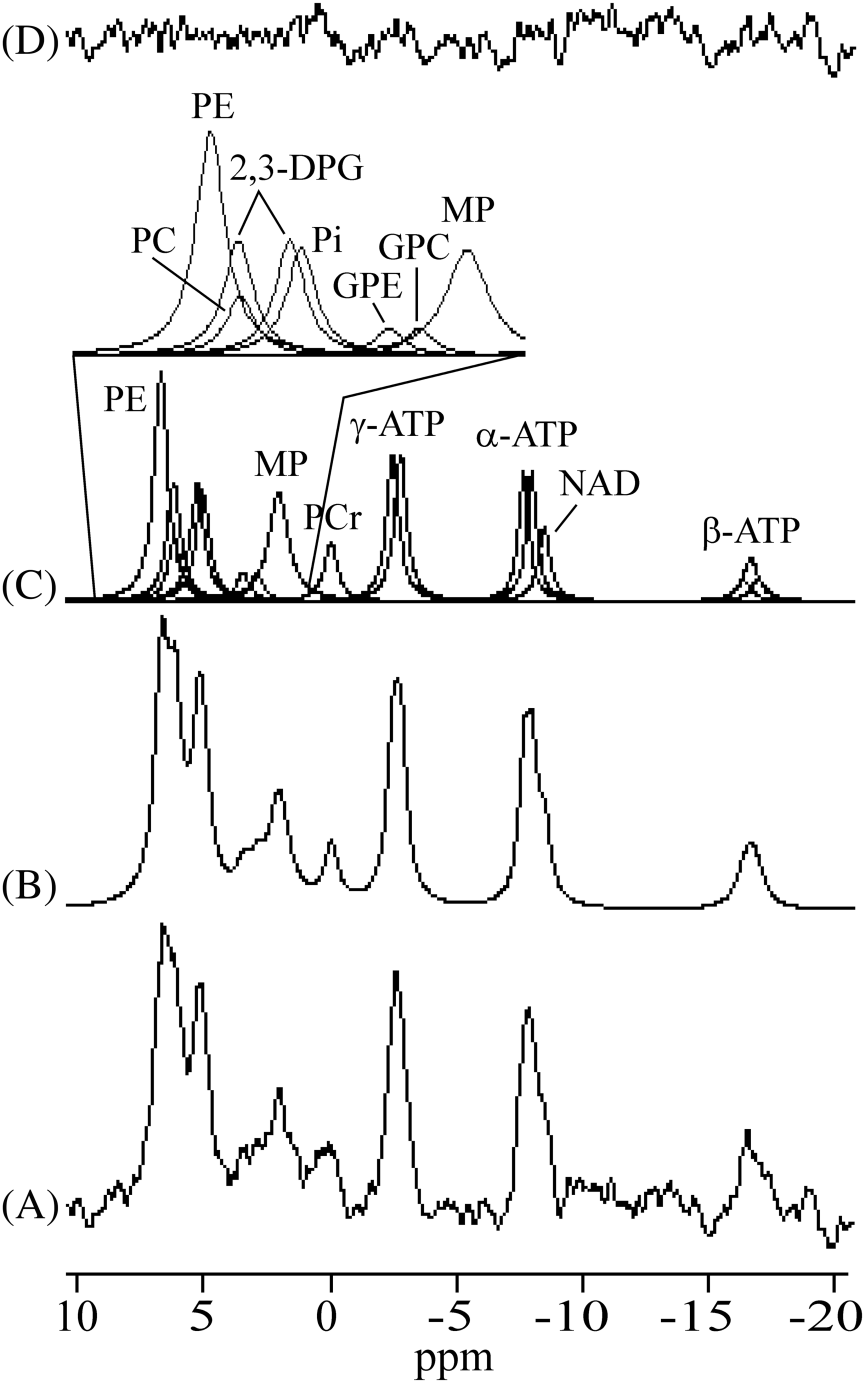
Summed spectrum of all volunteers (A), fitted spectrum (B), individual components (C), and residue (D). A Lorentzian apodization of the FIDs corresponding to 8 Hz line broadening was applied.

**FIGURE 4:**
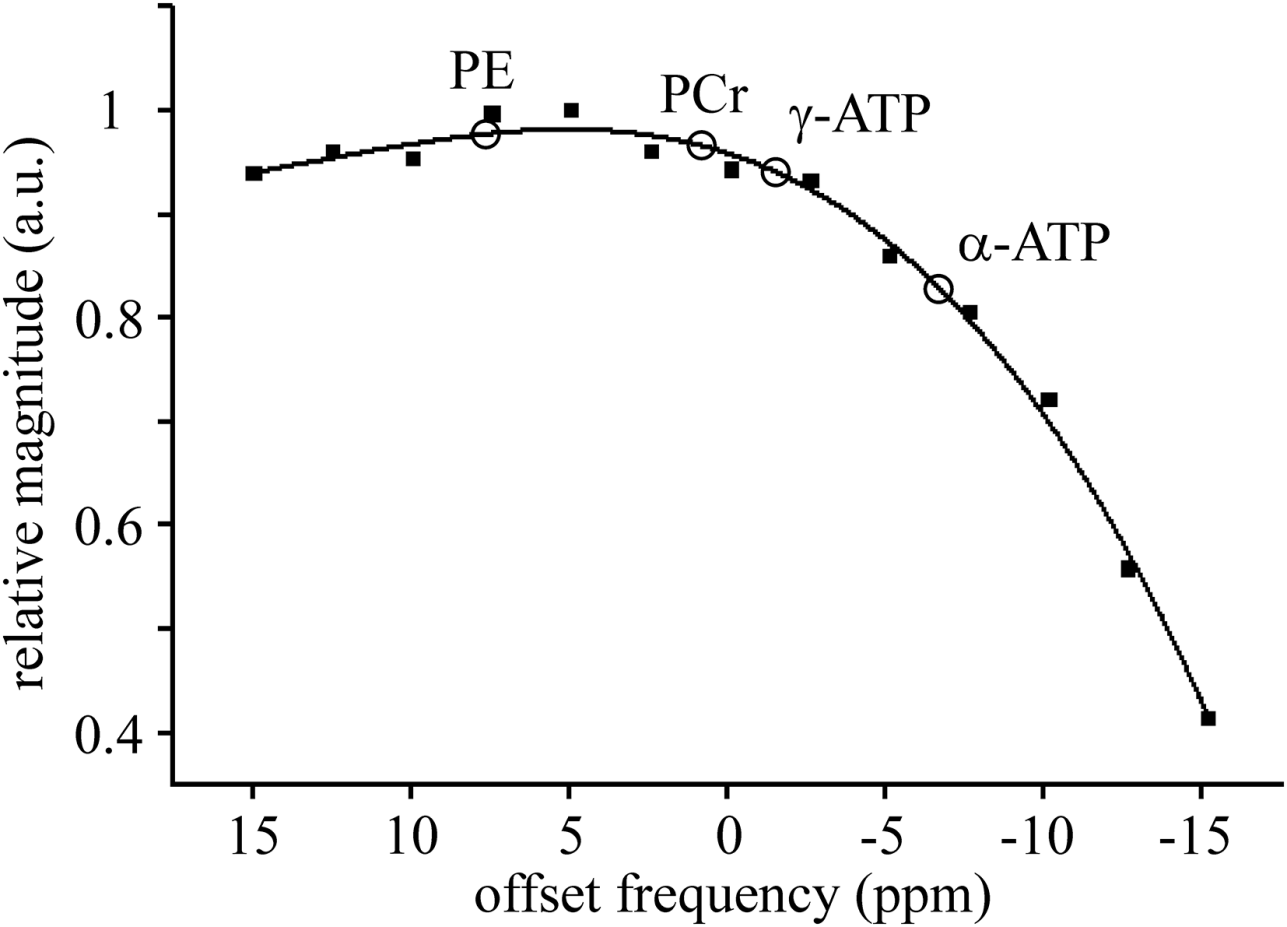
Excitation profile of the transmitter for a distance of 60 mm between the voxel centre and surface coil. Data were fitted by third order polynomial function (*R* ^2^ = 0.99).

A typical voxel size was 30 × 60 × 70 mm^3^ in the right–left (RL), feet–head (FH), and anterior–posterior (AP) directions, respectively, and this is depicted in Figure 1. The average voxel volume was 128.6 ± 40.7 cm^3^ (range 105–210 cm^3^) and the mean distance between the voxel centre and the surface coil was 57.4 ± 3.6 mm (range 52–62 mm). The full width at half maximum of the water line after shimming was 37 ± 7 Hz (range 30–45 Hz). The voxel position of the transmitter's carrier frequency, *f*_31P_, is depicted using the yellow rectangles in Figure 1. Carrier frequency was determined from the water resonance frequency *f*_0_ multiplied by the ratio of ^31^P and ^1^H gyromagnetic ratios, *γ*_31P_ and *γ*_1H_ (*f*_31P_ = *f*_0_ *γ*_31P_/*γ*_1H_). It is worth noting that the PCr line position (if present) is at approximately 0.86 ppm with respect to *f*_31P_. White rectangles depict voxel positions corresponding to PE, γ‐ATP, α‐ATP, and β‐ATP spectral lines. Chemical shift displacements (absolute values) of the PE, γ‐, α‐, and β‐ATP voxels (white rectangles in Figure 1) with respect to yellow rectangles (carrier frequency of the transmitter) were approximately 4 × 8.3 × 9.6, 1.6 × 3.2 × 3.6, 4.6 × 9.3 × 10.8, and 9.9 × 19.8 × 23.1 mm^3^ in RL, FH, and AP directions, respectively. The individual spectra of all volunteers are shown in Figure 2. The added spectrum and fitting results are shown in Figure 3. The small PCr signal intensity at 0 ppm reveals that contamination of the spectra from skeletal muscle in the chest wall was very small. Cramér–Rao lower bounds (CRLBs) of the total resulting spectrum (Figure 3) were 5%, 13%, and 4% for PE, MP, and γ‐ATP line fits, respectively. CRLBs of the same spectrum were more than 50% for PC, 2,3‐DPG, P_i_, GPE, and GPC. Mean CRLBs of the individual spectra (Figure 2) were 11.5 ± 2.8% (range 8–15) for PE; 29.2 ± 8.5% (range 16–39) for MP, and 8.8 ± 2.2% (range 6–12%) for γ‐ATP. Mean CRLBs were more than 40% for PC, 2,3‐DPG, P_i_, GPE, and GPC. The transmitter excitation profile for a 60 mm distance between the centre of the voxel and the surface coil is depicted in Figure 4. The spectral intensity fractions of PME+2‐DPG, 3‐DPG + P_i_, and PDE + MP and the spectral intensity ratios are shown in Tables 1 and 2, respectively.

**TABLE 1:** Spectral intensity fractions expressed as the fraction of summed PME, 2,3‐DPG, P_i_, PDE, MP, and γ‐ATP intensities

PME + 2‐DPG (%)	3‐DPG + P_i_ (%)	PDE + MP (%)	γ‐ATP (%)
36.7 ^a^	20.6 ^a^	19.2 ^a^	23.5 ^a^
36.4 ^a^ ^,^ ^b^	20.3 ^a^ ^,^ ^b^	19.0 ^a^ ^,^ ^b^	24.3 ^a^ ^,^ ^b^
36.5 ± 4.2 ^c^	21.2 ± 1.5 ^c^	18.8 ± 3.6 ^c^	23.5 ± 4.3 ^c^
36.1 ± 4.2 ^b^ ^,^ ^c^	21.0 ± 1.5 ^b^ ^,^ ^c^	18.6 ± 3.6 ^b^ ^,^ ^c^	24.3 ± 4.4 ^b^ ^,^ ^c^

^a^ Value acquired from the summed spectrum of all volunteers.

^b^ Value corrected for transmitter excitation profile.

^c^ Mean ± 1 SD computed from the individual spectra of the volunteers.

**TABLE 2:** Spectral intensity ratios

(PME + 2‐DPG)/ (3‐DPG + P_i_ )	(PDE + MP)/ (3‐DPG + P_i_ )	(PME+2‐DPG)/ γ‐ATP	(3‐DPG + P_i_ )/ γ‐ATP	(PDE + MP)/ γ‐ATP
1.79 ^a^	0.93 ^a^	1.56 ^a^	0.87 ^a^	0.82 ^a^
1.79 ^a^ ^,^ ^b^	0.94 ^a^ ^,^ ^b^	1.5 ^a^ ^,^ ^b^	0.84 ^a^ ^,^ ^b^	0.78 ^a^ ^,^ ^b^
1.73 ± 0.23 ^c^	0.88 ± 0.16 ^c^	1.61 ± 0.43 ^c^	0.94 ± 0.23 ^c^	0.83 ± 0.23 ^c^
1.73 ± 0.23 ^b^ ^,^ ^c^	0.89 ± 0.16 ^b^ ^,^ ^c^	1.54 ± 0.41 ^b^ ^,^ ^c^	0.9 ± 0.22 ^b^ ^,^ ^c^	0.79 ± 0.22 ^b^ ^,^ ^c^

^a^ Value acquired from the summed spectrum of all volunteers.

^b^ Value corrected for transmitter excitation profile.

^c^ Mean ± 1 SD computed from the individual spectra of the volunteers.

## 4 DISCUSSION

In this work, we have presented the first 3 T phosphorus spectra of the healthy human spleen in situ. To the best of our knowledge, this is the first report where spectra of the spleen were measured using a single voxel (3D‐ISIS) approach combined with ^1^H decoupling and NOE enhancement.

Previous phosphorus spectra of healthy spleen were measured on 1.5 T scanners.^15^, ^16^, ^17^ The VOI was defined using the sensitive volume of the transmit–receive coil together with 1D^15^ or 3D MRSI.^16^, ^17^ These spectra were partially contaminated from the surrounding tissues due to the sensitive volume of the surface coil or by signal bleeding from the neighbouring voxels.^14^ One clear disadvantage of spectroscopy at lower magnetic field strength is a worsened signal‐to‐noise ratio (SNR) compared with the higher field strengths. However, CSDEs at 1.5 T are much smaller compared with that at 3 T. CSDEs and excitation profile were neglected in the majority of studies reported at 1.5 T.

In this study, the single voxel approach was chosen considering the fact that the only available phosphorus transmit–receive surface coil had a diameter of 14 cm and the average normal adult spleen is less than 12 cm long, up to 7 cm wide, and 3–5 cm thick.^22^, ^23^ The slab selective methods were excluded based on these facts because the sensitive volume of the ^31^P surface coil exceeded the size of an average spleen. The main advantage of our single‐voxel approach is a reliable VOI selection. Despite this, CSDEs proved to be a major issue in our measurements. CSDE is directly proportional to the voxel size (‘slice’ thicknesses *D* of the voxel) and the chemical shift difference between spectral lines Δ*f* (Hz) of interest, and inversely proportional to the effective bandwidth of the excitation *B*_1_ pulses (CSDE = *D* Δ*f*/BW; BW, bandwidth). At magnetic field strength of 3 T or more, the maximum achievable intensity of excitation pulses is subject to tight limits. The maximum *B*_1_ must match the hardware limit defined for the transmit coil and permissible power deposition (specific absorption rate). Bandwidths of adiabatic excitation pulses at magnetic field strengths of 3 T or more are narrower than corresponding pulses at 1.5 T. We further note that the bandwidth of adiabatic inversion pulses can be increased up to 15–40 kHz with GOIA pulses,^4^ but unfortunately, GOIA pulses were not implemented in our sequence. Enlarged CSDEs in our study are a consequence of reduced bandwidth of *B*_1_ pulses, larger voxel sizes, and increased chemical shift differences Δ*f* between respective spectral lines. The phosphorus signal of ISIS sequences can also be contaminated from signal contributions caused by ‘*T*_1_ smearing’ from regions outside the voxel if *T*_R_ is shorter than 5*T*_1_. *T*_1_ smearing is a consequence of the susceptibility of excitation pulses to *B*_1_ variations.^4^ In this study the *T*_1_ smearing effect was reduced by using adiabatic excitations.

CSDEs together with a more pronounced decline of excitation profile caused substantial decrease of α‐ and β‐ATP intensities. Therefore, our quantitative evaluations were restricted to the spectral region from PE to γ‐ATP. We intentionally positioned PE and γ‐ATP voxels as close to the spleen as possible to minimize partial volume effects caused by CSDEs (Figure 1A and 1B) and signal contamination from surrounded tissue. We assumed that γ‐ATP intensity is an acceptable substitute for missing α‐ and β‐ATP lines in our quantifications, because in theory γ‐, α‐, and β‐ATP peaks have identical intensities.^24^ Differences between excitation profile corrected and uncorrected results were less than 1% (Table 1) because the positions of all evaluated spectral lines lie on the flat part of the excitation profile (Figure 4). The dependence on the excitation profile can be minimized further by shifting the *B*_1_ carrier frequency to about −250 Hz (4.8 ppm downfield), to the flatter region of the excitation profile (Figure 4). We did not utilize this shift to improve the intensity of α‐ATP, because a large part of an α‐ATP voxel can fall outside the spleen. It should also be noted that better SNR or shorter acquisition times could be achieved with patients suffering from splenomegaly because larger voxels can be chosen despite CSDEs.

Our spectral patterns, in both PME and PDE regions, did not agree with those reported in the first study by Smith et al.^15^ Their spectrum of normal spleen reveals large contamination from the surrounding muscle tissues (highest PCr peak), equal PME and P_i_ levels, and large PDE + MP signal intensity. The spectral interpretation inaccuracy of the authors was a consequence of poor spatial resolution and missing 2,3‐DPG intensities in their prior knowledge. However, our spectra agree well with the spectra reported by Negendank et al.^16^ and Arias‐Mendoza et al.^17^ Similar to their spectra, we also observed dominant 2‐DPG + PC and 3‐DPG + P_i_ intensities, but their intensities of PDE + MP peaks and ATP lines were smaller compared with our spectra. Higher ATP intensities in our spectra can be explained by the fact that, with broader lines and a higher magnetic field in our study, multiplets of ATP resonances collapse into singlets, resulting in higher apparent ATP peaks.

This work has several limitations. Our volunteer population was small, although comparable to or larger than the previous studies.^15^, ^16^, ^17^ The main limitation of our 3D‐ISIS sequence is the fact that the hyperbolic‐secant inversion and the detection *B*_1_ pulses have limited bandwidths and their respective amplitudes drop rapidly with distance from the surface coil. The consequences are large CSDEs and the excitation profile dependence on distance between the surface coil and the voxel. Another limitation is the fact that our quantitative results are only valid for our particular protocol and specific scanner because NOE enhancement depends on NOE method, NOE parameters, and type of hardware (MR scanner).

## 5 CONCLUSION

We have demonstrated that the localized ^31^P‐MRS of spleen in situ using single‐voxel ISIS is feasible on a 3 T scanner in a clinically acceptable acquisition time. Results should be corrected for transmitter excitation profile, and CSDEs need to be taken into consideration during placement of the VOI. Results presented here could potentially be used as a reference in future studies of splenomegaly caused by haematological malignancies.

## Data Availability

The data that support the findings of this study are available on request from the corresponding author.
